# New Books

**Published:** 2008-09

**A Question of Balance: Weighing the Options on Global Warming Policies**

William D. Nordhaus

New Haven, CT:Yale University Press, 2008. 256 pp. ISBN: 978-0-300-13748-4, $28

**Arsenic Pollution: A Global Synthesis**

Peter Ravenscroft, Hugh Brammer, Keith Richards

Hoboken, NJ:John Wiley & Sons, Inc., 2008. 640 pp. ISBN: 978-1-405-18601-8, $49.95

**Changes in the Human-Monsoon System of East Asia in the Context of Global Change**

Congbin Fu, J.R. Freney, J.W.B. Stewart, eds.

Hackensack, NJ:World Scientific Publishing Co., 2008. 384 pp. ISBN: 978-981-283-241-2, $88

**Climate Solutions: A Citizen’s Guide**

Peter Barnes

White River Junction, VT:Chelsea Green Publishing, 2008. 120 pp. ISBN: 978-1-6035-8005-2, $9.95

**Dire Predictions: Understanding Global Warming**

Michael E. Mann, Lee R. Kump

New York:DK Publishing, 2008. 208 pp. ISBN: 978-0-7566-3995-2, $25

**Emissions Trading: Institutional Design, Decision Making and Corporate Strategies**

Ralf Antes, Bernd Hansjürgens, Peter Letmathe, eds.

New York:Springer, 2008. 274 pp. ISBN: 978-0-387-73652-5, $119

**Energy and Climate Change: Creating a Sustainable Future**

David Coley

Hoboken, NJ:John Wiley & Sons, Inc., 2008. 672 pp. ISBN: 978-0-470-85313-9, $50

**Environmental Law, Policy, and Economics: Reclaiming the Environmental Agenda**

Nicholas A. Ashford, Charles C. Caldart

Cambridge, MA:MIT Press, 2008. 1,088 pp. ISBN: 978-0-262-01238-6, $90

**Global Warming, Natural Hazards, and Emergency Management**

George Haddow, Jane A. Bullock, Kim Haddow

Boca Raton, FL:CRC Press, 2008. 280 pp. ISBN: 978-1-420-08182-4, $59.95

**International Documents on Environmental Liability**

Hannes Descamps, Robin Slabbinck, Hubert Bocken

New York:Springer, 2008. 372 pp. ISBN: 978-1-4020-8366-2, $189

**Malformed Frogs: The Collapse of Aquatic Ecosystems**

Michael Lannoo

Berkeley:University of California Press, 2008. 288 pp. ISBN: 978-0-520-25588-3, $65

**Natural Climate Variability and Global Warming**

Rick Battarbee, Heather Binney, eds.

Hoboken, NJ:John Wiley & Sons, Inc., 2008. 288 pp. ISBN: 978-1-4051-5905-0, $95

**Not One Drop: Betrayal and Courage in the Wake of the Exxon Valdez Oil Spill**

Riki Ott

White River Junction, VT:Chelsea Green Publishing, 2008. 256 pp. ISBN: 978-1-933-39258-5, $21.95

**Potential Impacts of Climate Change on U.S. Transportation: Special Report 290**

Committee on Climate Change and U.S. Transportation, National Research Council

Washington, DC:National Academies Press, 2008. 296 pp. ISBN: 978-0-309-11306-9, $37

**Protecting Health in Europe from Climate Change**

B. Menne, F. Apfel, S. Kovats, F. Racioppi

Geneva:WHO Press, 2008. 51 pp. ISBN: 978-928-907187-1, $15

**Regulating Water and Sanitation for the Poor: Economic Regulation for Public and Private Partnerships**

Richard Franceys, Esther Gerlach

London:Earthscan, 2008. 320 pp. ISBN: 978-1-8440-7617-8, $97.50

**Retaking Rationality: How Cost Benefit Analysis Can Better Protect the Environment and Our Health**

Richard Revesz, Michael Livermore

New York:Oxford University Press, 2008. 262 pp. ISBN: 978-0-19-536857-4, $34.95

